# OMOP CDM Can Facilitate Data-Driven Studies for Cancer Prediction: A Systematic Review

**DOI:** 10.3390/ijms231911834

**Published:** 2022-10-05

**Authors:** Najia Ahmadi, Yuan Peng, Markus Wolfien, Michéle Zoch, Martin Sedlmayr

**Affiliations:** Institute for Medical Informatics and Biometry, Carl Gustav Carus Faculty of Medicine, Technische Universität Dresden, Fetscherstraße 74, 01307 Dresden, Germany

**Keywords:** OHDSI, OMOP CDM, EHR, PLP, prediction, machine learning

## Abstract

The current generation of sequencing technologies has led to significant advances in identifying novel disease-associated mutations and generated large amounts of data in a high-throughput manner. Such data in conjunction with clinical routine data are proven to be highly useful in deriving population-level and patient-level predictions, especially in the field of cancer precision medicine. However, data harmonization across multiple national and international clinical sites is an essential step for the assessment of events and outcomes associated with patients, which is currently not adequately addressed. The Observational Medical Outcomes Partnership (OMOP) Common Data Model (CDM) is an internationally established research data repository introduced by the Observational Health Data Science and Informatics (OHDSI) community to overcome this issue. To address the needs of cancer research, the genomic vocabulary extension was introduced in 2020 to support the standardization of subsequent data analysis. In this review, we evaluate the current potential of the OMOP CDM to be applicable in cancer prediction and how comprehensively the genomic vocabulary extension of the OMOP can serve current needs of AI-based predictions. For this, we systematically screened the literature for articles that use the OMOP CDM in predictive analyses in cancer and investigated the underlying predictive models/tools. Interestingly, we found 248 articles, of which most use the OMOP for harmonizing their data, but only 5 make use of predictive algorithms on OMOP-based data and fulfill our criteria. The studies present multicentric investigations, in which the OMOP played an essential role in discovering and optimizing machine learning (ML)-based models. Ultimately, the use of the OMOP CDM leads to standardized data-driven studies for multiple clinical sites and enables a more solid basis utilizing, e.g., ML models that can be reused and combined in early prediction, diagnosis, and improvement of personalized cancer care and biomarker discovery.

## 1. Introduction

Electronic health record (EHR) data have been used to store patient-specific information for decades, including structured data, such as diagnosis, medication, laboratory test results, and unstructured data obtained from clinical reports. Observational patient data are used in vast computational analyses, including the generation of individual patient profiles and detection of patient similarity based on clinical and genomics data [1]. With advancements in the field of genetics, it is possible to analyze large amounts of genomic data using different ML and other predictive methods that can widen the knowledge about diseases with a genetic background, such as cancer, as well as rare and unclear diseases. However, such algorithms need large patient cohorts to reach a clinical prediction scale and useful diagnostic decision support [2]. For this purpose, a harmonized research data repository is necessary to enable a joint analysis across institutions based on observational data [3].

The Observational Health Data Science and Informatics (OHDSI) initiative is a promising international effort to optimize secondary use of observational data by harmonizing and standardizing clinical data and to create scalable analytical tools [4,5]. The basis for this is the Observational Medical Outcomes Partnership (OMOP) Common Data Model (CDM), which ensures homogeneous storage of observational healthcare data across different databases with interoperable formats and standard terminologies. The terminologies for diagnoses/conditions, observations, and drugs within the OMOP CDM are based on, for example, International Classification of Diseases (ICD) codes [6], Systematized Nomenclature of Medicine – Clinical Terms (SNOMED-CT) [7], and normalized naming system for generic and branded drugs (RxNorm) [8]. To apply these concepts, one usually needs to retrieve the already mapped tables from the Automated Terminology Harmonization, Extraction and Normalization for Analysis (ATHENA) [9] standardized vocabulary tool from OHDSI. Afterwards, the harmonized data stored in the OMOP CDM format can be used in systematic studies, population-level estimations, drug and biomarker evaluations, as well as further patient-level prediction [10].

To develop tailor-made therapies for cancer patients, researchers must have access to genetic variants and their associated pathways together with the clinical information. Studies such as Unberath et al. [11] created a vocabulary set using the HUGO Gene Nomenclature Committee (HGNC) [12] for their specific use case, but a comprehensive standard vocabulary that can assist in sequencing data on the OMOP CDM and predictive modules in general would be essential.

The first attempt for this purpose was a Genomic Common Data Model (G-CDM) proposed in 2019 [13] to store next-generation sequencing (NGS) data. The G-CDM introduced four extension tables to the OMOP CDM which, to use any of the standardized OHDSI tools for the purpose of data analysis or prediction on data stored using this format, would require adoption of the tools, because they strictly follow the OMOP CDM structure. One important tool is named ATLAS – A unified interface for the OHDSI tools [14], which is an open-source web-based interface to configure analysis modules such as Patient Level Prediction (PLP) in the OMOP CDM [15,16]. In particular, PLP can be used to define Artificial Intelligence (AI)-based analyses on previously defined patient cohorts by using an easy-to-use graphical user interface. However, to tackle the challenge of enabling oncology data in the OMOP CDM without any structural changes, the OHDSI community has published the first version of a set of new vocabularies for presenting genomic data in the OMOP CDM in 2020 [17]. These new vocabularies are built based on different gene databases, e.g., ClinVar [18], Clinical Interpretation of Variants in Cancer (CIVic) [19], and Precision Oncology Knowledge Base (OncoKB) [20]. With the use of new vocabularies, the data can be represented in the OMOP CDM without the need for any further extensions. Additionally, an OMOP oncology module was introduced in 2021 [3], which extends the OMOP CDM and its terminologies to support the harmonized documentation of cancer conditions, treatment, and disease abstraction. This module uses the concepts from seven existing standards, namely, International Classification of Diseases for Oncology (ICD-O-3) [21], Hematology Oncology (HemOnc) [22,23], North American Association of Central Cancer Registries (NAACCR) [24], College of American Pathologists CAP [25], Nebraska Lexicon [26], National Cancer Institute (NCI) [27], and Anatomical Therapeutic Chemical (ATC) classification [28]. Moreover, the Radiology Common Data Model (R-CDM) for standardization of Digital Imaging Communications in Medicine (DICOM) was published in 2021 [29]. R-CDM uses the RadLex glossary, which contains 75,000 radiology terms to harmonize DICOM imaging data into two extended tables, radiology occurrence and radiology image, on the OMOP CDM.

Clinical integration of the OMOP CDM can pave the way back to patients through facilitating access to relevant data, enabling multicentric, multidatabase studies to enhance statistical power and transfer results across populations [30,31,32]. With the recent advancements in the field of medical informatics, many predictive algorithms are known and used in the field of oncology, which benefits largely from the use of such models in uncovering unknown information about the cause and course of certain types of cancer. For the purpose of this review, we looked for studies that have taken advantage of such predictive models to perform cancer-related analyses on an OMOP CDM and evaluate to what extent the genomic vocabulary extension of the OMOP can serve current needs of ML-based predictions.

### Research Questions

Since this review aims to evaluate the applicability and current potential of the OMOP CDM in cancer prediction, the research questions that guided this study are as follows:

1. To what level are predictive models used and integrated in cancer prediction on the OMOP CDM, and where does it require more attention?
2. Does the existing genomic vocabulary in the OMOP cover the needs for analysis of genomic data using, e.g., the ATLAS PLP module?
3. What tools (other than PLP) exist to support these predictive analyses on cancer data in the OMOP?

## 2. Results

**Figure 1:**
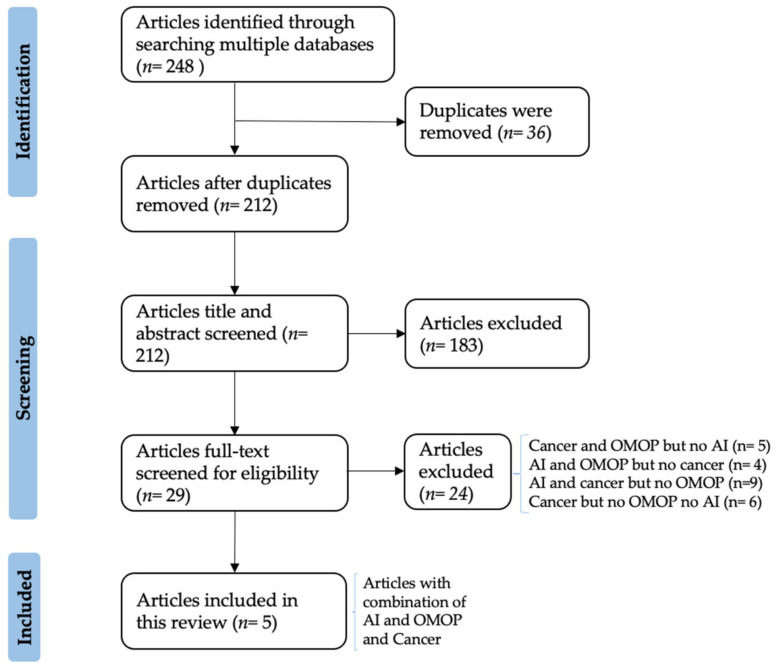
PRISMA Flow-chart diagram showing the paper selection process.

The literature screening resulted in 248 papers from 13 search engines, of which only five matched the scope of our review and are finally included (Figure 1). In particular, after duplicates’ removal, 212 articles were title- and abstract-screened. In this step, articles that did not indicate an AI-based prediction analysis and OMOP in their title or abstract were excluded.

Afterwards, the full text screening step analyzed in total 29 articles, out of which 15 were either focused on AI and cancer but without using the OMOP or focused solely on cancer or AI. The remaining nine articles either contained cancer studies on OMOP-based data not using predictive AI models [11,33,34,35,36] or performed predictive analysis on OMOP-based data of a non-cancerous disease [37,38,39,40,41,42,43,44,45]. An example for the first group are preliminary studies that are focused on harmonizing data in the OMOP using extract, load, and transform (ETL) processes. The articles that perform predictive analysis on other than cancerous data partially use different machine learning and deep learning methods. One of these studies is Hardin et al. [46] that uses the OHDSI PLP module for the development of predictive models. Since these excluded studies also contain a valuable source of information for the current review, detailed information of the most important excluded articles and the finally included five articles can be obtained in the attached Supplementary Table S1 (color-coded in grey). In the following, we highlight the studies that ultimately contain aspects of AI and the OMOP in the cancer domain.

Among the included papers, Felmeister et al. [1] focus on the pediatric rare brain tumor and follow an exploratory approach to extract pertinent information from a large simulated observational dataset based on the OMOP and discover data points that contribute to the data-driven phenotype of a diagnosed subject. An example of such a data point is population-based survival estimates. The authors apply a supervised prediction approach and take advantage of the Logistic Regression (LR), Linear Discriminant Analysis (LDA), K-Neighbors classifier (KNN), Decision Tree classifier (CART), Gaussian Naïve Bayes (NB), and Support Vector Machine (SVM) algorithms. The models are applied on a simulated cohort of 1000, in which KNN performs best with the highest percentage of correctly identified cases. SVM and LR are the second and third best-performing ML algorithms. The analysis shows that usage of OMOP CDM observational data in exploration analysis can lead to valuable discoveries.

Meystre et al. [47] train a Natural Language Processing (NLP) method using manually annotated physician letters for subsequent automatic detection of patient eligibility for breast cancer clinical trials. The authors encode the clinical trial eligibility criteria to the corresponding clinical information system. The clinical notes were stored in the notes table in the OMOP CDM. They use NLP and an SVM classifier method to extract the patient-derived EHR information from the existing free text notes written by physicians. The cohort was designed by using the ATLAS platform of OHDSI. The study shows that NLP is able to extract the eligibility criteria for clinical trials from EHR notes from a cohort of 229 patients, with an average recall and precision of 84.6% and 64.4%. In comparison, SVM models perform better with an average recall of 90.9% and precision of 89.7%. Using the extracted eligibility information, the patients were classified to determine eligibility using an SVM binary classifier with high accuracy.

Unlike Felmeister et al., the third study by Seneviratne et al. [48] uses tree-based classification models, such as Lasso Penalty (LASSO), Random Forest (RF), Gradient Boosted Machine (GBM), and Extreme Gradient Boosting (XGB), on a cohort with prostate cancer. The algorithms classify metastatic cancer from non-metastatic cases based on the stage of cancer, which is usually documented in text form in medical notes, which means it is only feasible to extract cancer stage information on population level, when an AI-based approach is used. The study demonstrates identification of patients with metastatic prostate cancer in a cohort of 5861 patients using an RF classifier with a precision and recall of 90% and 40%, respectively. The RF model outperforms other models, including normal ICD code search, which leads to a recall and precision of 54% and 33%.

Moreover, the Information Technology for the Future of Cancer (ITFoC) [49] introduces a framework for the validation of AI algorithms with omics and clinical data for prediction of the treatment response in triple-negative breast cancer (TNBC) [50]. In this framework, the AI models will be developed and validated on real-world data. The clinical and -omics data will be harmonized via the OMOP CDM and terminologies, such as ICD-10, Logical Observation Identifiers Names and Codes (LOINC), and SNOMED-CT.

Furthermore, Lee et al. [51] perform a retrospective study of data obtained from seven hospitals in Korea that adopted the OMOP CDM as main research data repository. The study aims to find the association of angiotensin-converting enzyme inhibitor (ACEi) and angiotensin receptor blocker (ARB) with lung cancer development. Similar to Meystre et al., for cohort definition and defining the baseline characteristics of the study, the OHDSI tool ATLAS was used.

As shown in Table 1, all of the abovementioned articles use the OMOP CDM as a data standardization model, transform their datasets to this format, and design their AI-based analysis on it. The vocabularies that are used in these papers to transform data into the OMOP CDM structure include the International Classification of Diseases Clinical Modification, 9th Revision (ICD-9-CM), International Classification of Diseases Clinical Modification, 10th Revision (ICD-10-CM), SNOMED-CT, and LOINC codes. Moreover, a wide range of models are used as predictive models in the aforementioned papers, starting from classical machine learning methods, e.g., RF, GBM, all the way to other regression and classification methods, including linear regression, lasso regression, SVM, and k-Nearest Neighbors (KNN). Since the OMOP CDM harmonizes different data structures, the same predictive tool or trained model can be applied in different medical studies.

In summary, all of the abovementioned finally included articles use the OMOP as a common data model, out of which two (Meystre et al. and Lee et al.) use ATLAS for the purpose of cohort definition. Seneviratne et al. use only tree-based methods, whereas the other studies commonly utilize a combination of different types of methods (e.g., tree-based, boosting, SVMs). The use of different methods can also be obtained by using the PLP from OHDSI. Only Felmeister et al. use Centers for Medicare and Medicaid (CMS) Medicare Claims Synthetic Public Use Files (SynPUF) simulated data [52], which is a freely available dataset converted to the OMOP CDM used for benchmarking studies and technology implementations. A single study (Meystre et al.) uses unstructured, i.e., free text data for the initial analysis, and Tsopra et al. uses -omics data in addition to structured clinical data.

## 3. Discussion

In the era of cancer precision medicine, the need for targeted treatment protocols is increasing, and only predictive analyses may provide comprehensive information [53,54,55].

The genomic vocabulary in the OMOP CDM is a step forward towards harmonization of genomic data, which has the potential to enable analyses on combined clinical and sequencing data. However, three of the articles included in this review were published before 2020, meaning that they do not use the genomic vocabularies in the OMOP CDM but rather limit their features to clinical data. Lee et al. 2021 [51] do not mention a use of genomic vocabulary, but in the framework introduced by Tsopra et al. [50], the clinical and -omics data for TNBC will be harmonized on the OMOP CDM. Therefore, although the OMOP has enabled successful large-scale AI-based studies for some of the included articles, so far, there is no explicit evidence of how predictive methods can be applied to real-world oncological data represented in the OMOP CDM via genomics vocabularies. Moreover, a use of PLP is recommended by the OHDSI community also for genomic data analyses because this way, it conforms with standard practices. Currently, PLP is not mentioned in any of the articles that are in this review and focuses on cancer analysis on the OMOP. PLP is designed to support the clinical decision-making process based on the available individual medical history of the patient. The methods suggested in the articles in Table 1 are tested on the dataset by the authors and are not evaluated in routine care. Interestingly, most of the AI algorithms used in the articles (e.g., RF, KNN, and SVM) are included in the current version of PLP (https://github.com/OHDSI/PatientLevelPrediction/tree/main/R (accessed on 7 July 2022). Additionally, the PLP module also supports a set of deep learning methods, such as convolutional neural networks and recurrent neural networks, but we did not observe these methods in the included studies. This is an indication that most of the existing analyses might be replaced by the standard OHDSI tool to answer the underlying research questions [15,16]. This would improve the reusability and transparency of multicentric studies in general. However, it is also possible to customize AI-based prediction analyses for OMOP CDMs with individual programming languages and packages in R or Python, which allows for a more individualized analysis, if desired. An indication of the low numbers of published manuscripts that are currently available could be attributed to the ongoing multiple standardization efforts within OHDSI, which are highly dependent on currently developed infrastructures and implementations, such as PLP and genomic vocabulary of the OMOP.

It is worth mentioning that while our search string suffices as regards our research question (OMOP CDM’s ability concerning cancer prediction), it did not include the words “AI” and “oncology”, and a broader search string, not limited to only “cancer”, may possibly lead to different results. Interestingly, there are multiple international consortia arising built upon the OMOP. In particular, the Harmony Alliance recently presented a prime example of a European-wide OMOP-CDM-based study, in which an ML-based tool was introduced to predict the risk of relapse after first remission in leukemia patients [31]. The Data Analytics and Real World Interrogation Network (DARWIN EU) is aiming to deliver timely and reliable evidence by use of OMOP-based real-world data on disease and patient population, and the use, safety, and effectiveness of medicines, including vaccines, throughout the lifecycle of a medicinal product [56,57]. Moreover, the European Network of Excellence for Big Data in Prostate Cancer (PIONEER) performs predictive analysis on OMOP-based patient data [58,59]. These large-scale research studies likewise demonstrate the current need and versatile application scenarios for more envisaged OMOP-CDM-based studies in the future.

## 4. Materials and Methods

This paper follows the guidelines of the PRISMA extension for scoping reviews (PRISMA-ScR) [60]. According to the research questions stated above, we have the following search string: “cancer AND ((machine learning) OR (prediction) OR (algorithm)) AND (OHDSI) OR (OMOP)”.

### 4.1. Paper Identification

We conducted a systematic literature search for articles published between 2016 and 2021 in the most relevant databases in the domain, namely, PubMed, BMC, JAMIA, Journal of Bioinformatics, PLOS ONE, Hindawi, BMC Medical Informatics and Decision Making, Elsevier, Sage, Springer, Science Direct, Nature, and IEEE, with our search string. The article search was conducted on 27 April 2022. The results of our article search are processed within a library in the Zotero Citation Manager [61].

### 4.2. Paper Inclusion and Exclusion Criteria

The inclusion and exclusion criteria used in this study are shown in Table 2.

### 4.3. Selection and Review of Articles

After duplicate removal by the built-in function of Zotero, the study selection process was performed in two steps in accordance with the criteria from the Table 2**.** Two authors (NA, YP) independently performed a title and abstract screening and discussed the conflicts. In the second step, both authors (NA, YP) performed a full-text screening on the articles and resolved the conflicts after discussion.

### 4.4. Data Charting

After identifying relevant articles, an extraction table with a focus on the use of the OMOP CDM, the utilized predictive methods, and the terminologies used was created by NA and approved by YP. The data extraction was carried out by NA and approved by YP.

## 5. Conclusions

In line with the recently published perspective by Rehm et al. 2021 [62], we see the implementation of standards and frameworks for clinical data sharing as a critical step to advancing genomic medicine. In particular, the OMOP CDM has the potential to enable international collaborative analyses, which is a significant component for cancer precision medicine. Using the comprehensive genomics vocabularies, oncology data can be harmonized in the OMOP CDM, and this can lead to advancements in this field. Moreover, given that these vocabularies enable mapping and transferring oncology data into the OMOP CDM, the analysis of those data on a population level is now more possible than ever before.

Since the genomic vocabulary on the OMOP CDM and the R-CDM was recently published, these can be seen as a first, initial step that paves the way for mapping and transferring oncology data into the OMOP CDM. However, most of the articles included in this review were published before the release of the genomic vocabularies for the OMOP CDM. Therefore, a follow-up study needs to be conducted to obtain a measurable impact about how comprehensively the genomic vocabularies add up to existing AI models’ accuracy within the field of cancer precision medicine.

In addition, a broad use and continuous development of standards, especially within the field of oncology research, is essential to fully utilize the benefits of data harmonization across different cancer care entities. The use of AI-based approaches, such as NLP, SVM, and RF, has proven efficient in identification/classification of patients with certain characteristics and led to an improvement in the cohort identification process for clinical trials and other observational research [47,48]. In brief, standardized data, represented, for example, as the OMOP CDM, can serve as a solid base to enable a decentralized use of AI models that is needed for an optimized analysis on population-level estimation, patient-level prediction, and more specifically in cancer survival, time-based analysis, and biomarker discovery [1].

This review provides a first hallmark into current applications and the usefulness of the OMOP CDM for AI-based cancer prediction and likewise summarizes and promotes the beneficial use of OMOP CDMs in cancer prediction. In prospective future works, we will evaluate the application of PLP in ATLAS, as a predictive AI framework, for the purpose of cancer precision medicine in the OMOP CDM with the genomic vocabularies [17]. This focus, which is beyond the current study, will investigate whether existing modules in PLP are able to handle real-world oncological datasets using genomics vocabularies.

## Figures and Tables

**Table 1:** An overview of the dataset size and features used in the articles, vocabularies used to transform them into OMOP CDM format, and predictive models used to analyze the data.

Article	Dataset Size	Features	Vocabularies	Predictive Models
Felmeister et al. 2017 [1]	1000 Patients	patient, condition, observation, drug exposure and demographics (gender, race, date of birth, etc.)	ICD-9-CM, ICD-10-CM, SNOMED-CT	LR, LDA, KNN, CART, NB, and SVM
Meystre et al. 2019 [47]	229 Patients	patient identifier, gender, date of birth, height, weight, diagnostic code, procedure code, and clinical notes	LOINC, SNOMED-CT	NLP and SVM
Seneviratne et al. 2018 [48]	5861 Patients	conditions, procedures, medications, observations, and laboratory values	ICD-9 and ICD-10	LASSO, RF, GBM, and XGB
Tsopra et al. 2021 [50]	-	-	ICD-10, LOINC, and SNOMED-CT	-
Lee et al. 2021 [51]	207,794 Patients	age group, medical history: general (e.g., dementia, cardiovascular disease (e.g., arterial fibrillation), and neoplasms (e.g., malignant neoplasm of anorectum)	-	Cox regression

**Table 2:** Inclusion and exclusion criteria for the title and abstract screening and full-text screening.

Screening Round	Inclusion	Exclusion
Title and abstract screening	The article is primary research in a peer-reviewed journal or conference.	The article is of any other type, for instance, study protocols, commentaries and editorials, tutorials, project reports, medical case studies, and master and doctoral thesis.
	The article is written in English.	The article is written in a language other than English.
	The title or abstract mention analysis of cancer data.	The title or abstract do not mention analysis of cancer data.
	The title or abstract mention OMOP or OHDSI.	The title or abstract do not mention OMOP or OHDSI.
Full-text screening	The article allows open access to full text.	The article does not allow open access to full text.
	The article defines a predictive approach for cancer medicine.	The article defines a predictive approach but for a domain other than cancer medicine
	The predictive approach in the article uses the OMOP CDM as the data model.	The predictive approach in the article does not use the OMOP CDM as the data model.

## Data Availability

Not applicable.

## Supplementary Materials

The following supporting information can be downloaded at: https://www.mdpi.com/article/10.3390/ijms231911834/s1.
